# Inhibition of caspase-9 aggravates acute liver injury through suppression of cytoprotective autophagy

**DOI:** 10.1038/srep32447

**Published:** 2016-09-01

**Authors:** Rui Guo, Bin Lin, Jing Fei Pan, Emily C. Liong, Ai Min Xu, Moussa Youdim, Man Lung Fung, Kwok Fai So, George L. Tipoe

**Affiliations:** 1 School of Biomedical Sciences, LKS Faculty of Medicine, The University of Hong Kong, SAR, Hong Kong; 2 School of Optometry, Faculty of Health and Social Sciences, Hong Kong Polytechnic University, SAR, Hong Kong; 3 Brain Hormone Healthy Aging Centre, LKS Faculty of Medicine, The University of Hong Kong, SAR, Hong Kong; 4 Department of Medicine, LKS Faculty of Medicine, The University of Hong Kong, SAR, Hong Kong; 5 Faculty of Medicine, Technion-Israel Institute of Technology, Haifa, Israel

## Abstract

Acute liver disease is characterized by inflammation, oxidative stress and necrosis, which can greatly influence the long term clinical outcome and lead to liver failure or cancer. Here, we initially demonstrated the beneficial role of caspase-9-dependent autophagy in acute liver injury. Treatment with caspase-9 inhibitor z-LEHD-FMK in HepG2 cells, AML12 cells and C57BL/b6N mice exacerbated CCl_4_-induced acute hepatocellular damage, and also down-regulated autophagy markers expression levels, indicating that caspase-9 inhibition may aggravate acute liver damage by suppressing cytoprotective autophagy. CCl_4_ was used as an acute liver injury inducer which caused oxidative stress and apoptosis through up-regulation of HIF-1α, as well as triggered hepatic inflammation and necroptosis via TLR4/NF-κB pathway. Caspase-9 Thr125 site was firstly phosphorylated by ERK1/2 which subsequently activated the cytoprotective autophagy process to attenuate acute CCl_4_ injury. Caspase-9 inhibition further aggravated hepatic necroptosis through NF-κB expression, leading to increased pro-inflammatory mediators levels, suggesting a protective role of caspase-9-dependent autophagy in the inflammatory process as well as its possibility being a new therapeutic target for the treatment of acute liver injury.

Acute and chronic liver diseases are characterized by hepatic inflammation, oxidative stress and apoptosis. These underlying events greatly influence the long term clinical outcome which can lead to liver failure or cancer^1^. Any forms of treatment that can reduce these critical events possess great promise in the clinical management of liver diseases.

The acute liver injury model of carbon tetrachloride (CCl_4_) on liver is well established. Injection with CCl_4_ significantly enhances oxidative stress, hepatic inflammation, cellular apoptosis, necrosis, fibrosis, and even liver cancer in mice^2^. A great number of researchers have demonstrated the mechanisms of CCl_4_ toxicity in the liver. Once CCl_4_ is injected, the Cytochrome P-450 2E1 (CYP2E1) firstly catalyzes it into trichloromethyl free radical (CCl_3_*), which finally combines with oxygen to generate even more reactive trichloromethyl peroxyl radical (CCl_3_OO*)^3^. As a result, these reactive oxygen species (ROS) can cause hepatic oxidative stress, apoptosis, inflammation and fibrosis, which ultimately contribute to further cell damage and death.

Autophagy has been demonstrated to play a protective role in a number of liver injury models. Zhou reported that enhancing autophagy significantly decreases lipid accumulation in steatotic L-02 cells^4^. In addition, Rautou has shown that autophagy fights to keep cells alive under stressful “life-threatening” conditions in acute liver injury^5^. The expression pattern of caspase-9 is also similar with that of autophagy marker Beclin1^6^, suggesting that caspase-9 is likely to be involved in the autophagic process. To investigate the role of caspase-9, Zuo has demonstrated that ROS contributed to caspase-9 modification^7^, indicating that caspase-9 may participate in oxidative stress-related autophagic process.

M30 is a multifunctional non-toxic and neuroprotective compound with MAO-A and B inhibitory activity, which combines the antioxidant chelator moiety of an 8-hydroxyquinoline derivative of the brain permeable iron chelator VK28 and the propargyl moiety of the anti-Parkinsonian MAO-B inhibitor rasagiline^8^. It reduces H_2_O_2_-triggered oxidative stress by enhancing the expression of antioxidant enzymes in insulin-producing β-cells, indicating its antioxidant property^9^. Additionally, it can also protect the liver against ethanol-mediated injury^10^.

In this study, multifunctional M30 served as a therapeutic compound which was given to human HepG2 cells, AML12 cells and C57BL/b6N mice, in order to demonstrate the possibility of any underlying role of caspase-9 in the cytoprotective autophagic process in an acute liver injury model. The effect of caspase-9 phosphorylation on liver inflammation involving the inhibition of TLR4 has also been investigated.

## Methods

### Reagents

M30 pure powder was kindly provided by Prof Youdim (Eve Topf Centre of Excellence for Neurodegenerative Diseases, Technion-Rappaport Faculty of Medicine, Israel). Carbon tetrachloride was purchased from Tianjin Baishi Chemical (Tianjin, China). Phosphatase inhibitors 3-(4.5-dimethylthiazol-2-yl)- 2,5-diphenyltetrazolium bromide (MTT), chloroquine and necrostatin-1 were purchased from Sigma-Aldrich. Caspase-9 inhibitor (z-LEHD-FMK) was purchased from BD Biosciences (San Diego, CA, USA). Rapamycin was purchased from Calbiochem (Darmstadt, Germany). PD98059 was purchased from Cell Signaling (Danvers, MA, USA). Rabbit anti- Cytochrome P450 2E1 (CYP2E1) polyclonal antibody was obtained from Millipore (Billerica, MA, USA). Antibodies against, hypoxia-inducible factor 1 alpha (HIF-1α), total IκB-α, Receptor interacting protein 3 (RIP3) were obtained from Santa Cruz Biotechnology (Santa Cruz, CA, USA). Antibodies of Beclin1, lysosome-associated membrane protein 1 (LAMP1), ATG5, caspase-9, and cleaved poly (ADP-ribose) polymerase (PARP), Receptor interacting protein 1 (RIP1), phospho-ERK, and total ERK were purchased from Cell Signaling (Danvers, MA, USA). Phospho-caspase-9 (Thr125) antibody was purchased from LSBio (Seattle, USA). LC3 antibody was purchased from Sigma-Aldrich (St. Louis, MO, USA). Fluorescein isothiocyanate (FITC)-conjugated AffiniPure goat anti-rabbit was bought from Jackson Company (ImmunoResearch Laboratories, Inc. USA).

### Cell culture and viability assay

HepG2 cells were grown in DMEM supplemented with 10% heat-inactivated fetal bovine serum (FBS) and 100 U/ml penicillin/streptomycin. In addition, AML12 mouse hepatocytes were cultured in 1:1 mixture of Dulbecco’s modified Eagle’s medium and Ham’s F12 medium with 0.005 mg/ml insulin, 0.005 mg/ml transferrin, 5 ng/ml selenium, and 40 ng/ml dexamethasone, as well as 10% FBS. Caspase-9 inhibitor z-LEHD-FMK (20 μM), autophagy activator rapamycin (20 nM), autophagy inhibitor chloroquine (20 μM), p-ERK1/2 inhibitor PD98059 (20 μM), or necroptosis inhibitor Necrostatin-1 (50 μM) was added to the medium of cultured cells 2 h before the addition of M30 (5 μM). The cultures were then further incubated for another 2 h before CCl_4_ was added to a final concentration of 2 μl/ml and incubated for another 1 h. Here, we pre-treated M30 to the system because CCl_4_ is a strong toxin which can cause a large number of cell death in a short period of time. The cells were divided into several dishes namely: control, CCl_4_ only, M30 only, CCl_4_+M30, combination of z-LEHD-FMK, Rapamycin, Chloroquine, PD98059, or Necrostatin-1, (z-LEHD-FMK, Rapamycin, Chloroquine, PD98059, or Necrostatin-1)+CCl_4_, and (z-LEHD-FMK, Rapamycin, Chloroquine, PD98059, or Necrostatin-1)+CCl_4_+M30. After the cells were harvested, cell viability was determined by colorimetric MTT assay based on conversion of MTT to blue formazan crystals by viable cells.

### Animal experiments

Healthy 8–10 weeks male and female C57BL/b6N mice were kept under standard condition in compliance with the requirement of The University of Hong Kong with free access to animal chow diet and tap water. The experimental protocols of this work followed the guidelines and regulations of our Ethics Committee and were approved by the Committee of Animal Use for Research and Teaching at The University of Hong Kong. The Laboratory Animal Unit of The University of Hong Kong is fully accredited by the Association for Assessment and Accreditation of Laboratory Animal Care International (AAALAC international). Before treatment, mice were kept under standard conditions for 1 week with free access to chow and tap water. Mice were divided into six groups (n = 6 per group; with equal number of males and females) namely: (1) control with vehicle administration (normal saline and olive oil); (2) CCl_4_ treatment (75 μl/kg in olive oil; intraperitoneal injection); (3) M30 treatment (5 mg/kg; intraperitoneal injection); (4) CCl_4_ and M30 post-treatment; (5) pre-treatment z-LEHD-FMK (Caspase-9 inhibitor; 3 mg/kg; intraperitoneal injection) with CCl_4_; (6) z-LEHD-FMK pre-treatment, CCl_4_ and M30 post-treatment; rapamycin treatment (10 mg/kg; intraperitoneal injection for 24 h). z-LEHD-FMK was intraperitoneally injected 2 h before the CCl_4_ injection, while M30 was injected 1 h after the CCl_4_ treatment. After 8 h CCl_4_ treatment, mice were euthanized by overdose of anesthesia. Blood samples and liver tissues were collected for further analyses.

### Tissue and blood samples processing and histological analysis

Serum samples were collected by centrifugation of whole blood samples at 1000 × g for 10 min at 4 °C and stored at −80 °C until use. Liver tissues were fixed in 10% phosphate-buffered formalin for 72 h, processed histologically, embedded in paraffin blocks, cut to 5 μm sections, and stained with hematoxylin and eosin (H&E staining).

### Serum alanine aminotransferase (ALT) assay

To evaluate the hepatic injury at the enzymatic level, serum ALT level was measured by using an ALT (SGPT) reagent kit (Teco diagnostics, Anaheim, CA, USA) according to manufacturer’s instructions.

### Enzyme-linked immunosorbent assay (ELISA) assay

Human TNF-α and NF-κB were tested utilizing TNF-α and NF-κB Human Ultrasensitive ELISA Kits (Life technologies, CA, USA), and mouse TNF-α and IL-1β were examined using the ELISA Kits from R&D systems (Minnesota, USA). For NF-κB assay, nuclear and cytoplasmic extracts were prepared and subjected to an enzyme-linked immunosorbent assay according to the manufacturer’s instructions. Then, the active NF-κB for mouse and human were assessed by the level of p65 in the nuclear fractions using an NF-κB/p65-active ELISA kit (Imgenex, San Diego, CA, USA) and NF-κB Human Ultrasensitive ELISA Kit (Life technologies, CA, USA), respectively.

### Measurement of malondialdehyde (MDA) level

The malondialdehyde levels were determined using a Bioxytech LPO-586TM kit (Oxis Research, Portland, OR, USA) according to manufacturer’s instructions. The reaction product was measured spectrophotometrically at 586 nm. Standard curves were constructed using 1,1,3,3-tetraethoxypropane as a standard. The MDA levels were normalized with corresponding protein amounts determined by a Bio-Rad Protein Assay Kit (Bio-Rad, Hercules, CA, USA) and expressed as percentage against the control level.

### Knockdown of TLR4 expression by small interfering RNA

HepG2 Cells were incubated in the plates with culture medium containing serum under normal growth conditions (typically 37 °C and 5% CO_2_) for 24 hr. The next day, cells were transfected with human TLR4 siRNA directed against TLR4 (ON-TARGETplus siRNA Reagents, Dharmacon, CO, USA) using Lipofectamine 2000 reagent (Invitrogen, Paisley, UK) according to the manufacturer’s protocol. Scramble siRNA was used as a negative control.

### TUNEL assay

The terminal deoxynucleotidyl transferase-mediated dUTP-nick end labeling (TUNEL) assay was utilized to demonstrate the apoptotic cell death in the liver, which detects 3′ hydroxyl ends in fragmented DNA as an early event in apoptotic cascade. The staining was performed according to the manufacturer’s instructions using the *in situ* cell death detection kit, (TUNEL assay, AP. Roche, IN, USA). DNase I recombinant was used as the positive control. The positive immunostained cells of the TUNEL assay were quantified and expressed as percent of control using a light microscope (Zeiss Axiolab, Carl Zeiss Inc. Germany).

### Immunofluorescence staining

Frozen sections were blocked with the solution containing 1% bovine serum albumin, 4% normal goat serum and 1% Triton X-100 for 2 h at room temperature. Then, they were incubated with anti-LC3II primary antibody with 1:50 dilution at 4 °C overnight. After washing for three times with 0.1 M PBS containing 0.5% Triton X-100 for 10 min each time, the sections were incubated with FITC with 1:500 dilution at room temperature for 2 h before examining them with a fluorescent microscope adopted with a DC 200 digital camera (Leica Microsystems Ltd., Heerbrugg, Switzerland). Then, the positive fluorescent stained cells were quantified and expressed as percent of control.

### RNA extraction and quantitative reverse-transcription polymerase chain reaction (Realtime-PCR)

Total RNA was extracted using illustraTM RNAspin mini kit (GE Healthcare, UK) and then reverse-transcribed using SuperScriptTM First-Strand Synthesis System (Invitrogen, Calsbad, CA, USA). The mRNA expression levels were measured using Takara SYBR premix Taq quantitative PCR system (Takara Bio Inc, Shiga, Japan) and MyiQ2 real-time PCR machine (Bio-Rad). The sequences for the specific primers are listed in Supplementary Table 1. Parallel amplification of GAPDH was used as the internal control. Relative quantification was done by using the 2^−△△Ct^ method. The relative expression of the specific gene to the internal control was obtained and then expressed as a percentage of the control value in the figures. All quantitative PCR procedures including the design of primers, validation of PCR environment and quantification methods were performed according the MIQE guideline^11^,^12^.

### Western blot analysis

Cytosolic protein was extracted by using NE-PER protein extraction system (Pierce Biotechnology, Rockford, IL, USA) with the addition of Halt phosphatase inhibitor cocktail (Pierce). Western blotting was performed as previously described^10^. The ratio of the optical density of the protein product to the internal control was obtained and was normalized as a percentage of the control value in the figures by Image J.

### Statistical analyses

Data from each group were pooled to generate a mean and standard deviation (SD). The normality of data distribution was examined by chi-square test, and comparisons were performed using One-way ANOVA followed by post-hoc Duncan test. All statistical analyses were performed using a SPSS software.

## Results

### Blocking caspase-9 remarkably inhibited autophagy and aggravated CCl_4_-induced cellular injury and hepatic necrosis

**Figure 1:**
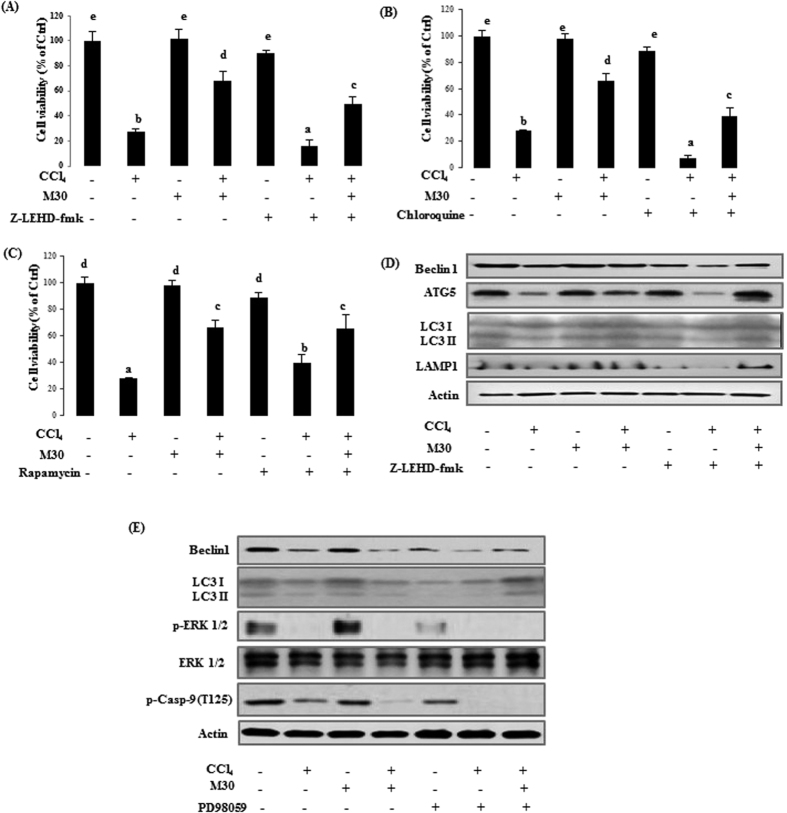
Inhibition of caspase-9 significantly inhibited autophagy and aggravated CCl_4_-triggered cellular damage in HepG2 cells. HepG2 cellular viability on Control, CCl_4_ (2 μl/ml), M30 (5 μM), and CCl_4_ + M30, and co-treatment with z-IETD-FMK (20 μM) (**A**); chloroquine (20 μM) (**B**); and rapamycin (20 nM) (**C**) groups. Beclin 1, ATG5, LC3II and LAMP1 protein expression levels were evaluated after treating z-IETD-FMK (20 μM) (**D**). The expressions of Beclin1, LC3II, p-ERK1/2, total ERK1/2, p-cas9 (Thr125) were also measured after adding PD98059 (20 μM) (**E**). Data presented are expressed as Mean ± SD (n = 6) and experimental groups marked by different letters represent statistical significant differences between groups at *p* *<* *0.05* (e.g. a and b mean a statistical significant difference between each other).

In cultured HepG2 cells, CCl_4_ caused a dose-dependent cell death of 10% at a concentration of 1 μl/ml, 80% at 2 μl/ml, and 95% at 4 μl/ml (data not shown). And, we selected the concentration at 2 μl/ml in this system. For *in vitro* study, M30 (5 μM) significantly enhanced the cell viability up to 60% in the co-treatment group when compared with 2 ul/ml of CCl_4_ alone. Co-treatment with autophagy inhibitor chloroquine (20 μM), and caspase-9 inhibitor Z-LEHD-fmk (20 μM) further decreased cell viability (Fig. 1A,B). On the contrary, Fig. 1C showed the opposite effect after adding the autophagy activator rapamycin (20 nM). CCl_4_ significantly down-regulated the Beclin1, ATG5, LC3II and LAMP1 protein expressions, which was reversed by M30, suggesting a protective role of autophagy induced by M30. Caspase-9 inhibitor z-IETD-FMK further down-regulated Beclin1, ATG5, LC3II and LAMP1 protein expressions when co-treated with CCl_4_, while inclusion of M30 markedly enhanced their expressions (Fig. 1D), suggesting that caspase-9 inhibition further suppressed the cytoprotective autophagy, which was previously enhanced by M30.

To investigate the signaling pathway of caspase-9 in autophagy, a major phosphorylation site Thr125 in caspase-9 was tested by using a mitogen-activated extracellular signal-regulated kinase (ERK) to induce cytoprotective autophagy and inhibit apoptotic process in response to a stimulus such as CCl_4_. As shown in Fig. 1E, treatment with CCl_4_ to HepG2 cells significantly reduced phospho-caspase-9 (Thr125) level, and a similar trend was also observed in p-ERK1/2 expression. Co-treatment with p-ERK1/2 inhibitor PD98059 (20 μM) further down-regulated both phospho-caspase-9 (Thr125) and p-ERK1/2 levels. This finding suggested that caspase-9 was phosphorylated by the upstream p-ERK1/2 in Thr125 site. Interestingly, the autophagy markers Beclin1 and LC3II also exhibited similar trend with phospho-caspase-9 (Thr125). Overall, our data showed that the cytoprotective autophagy in this system can be partly achieved through phosphorylation of caspase-9 Thr125 site.

**Figure 2:**
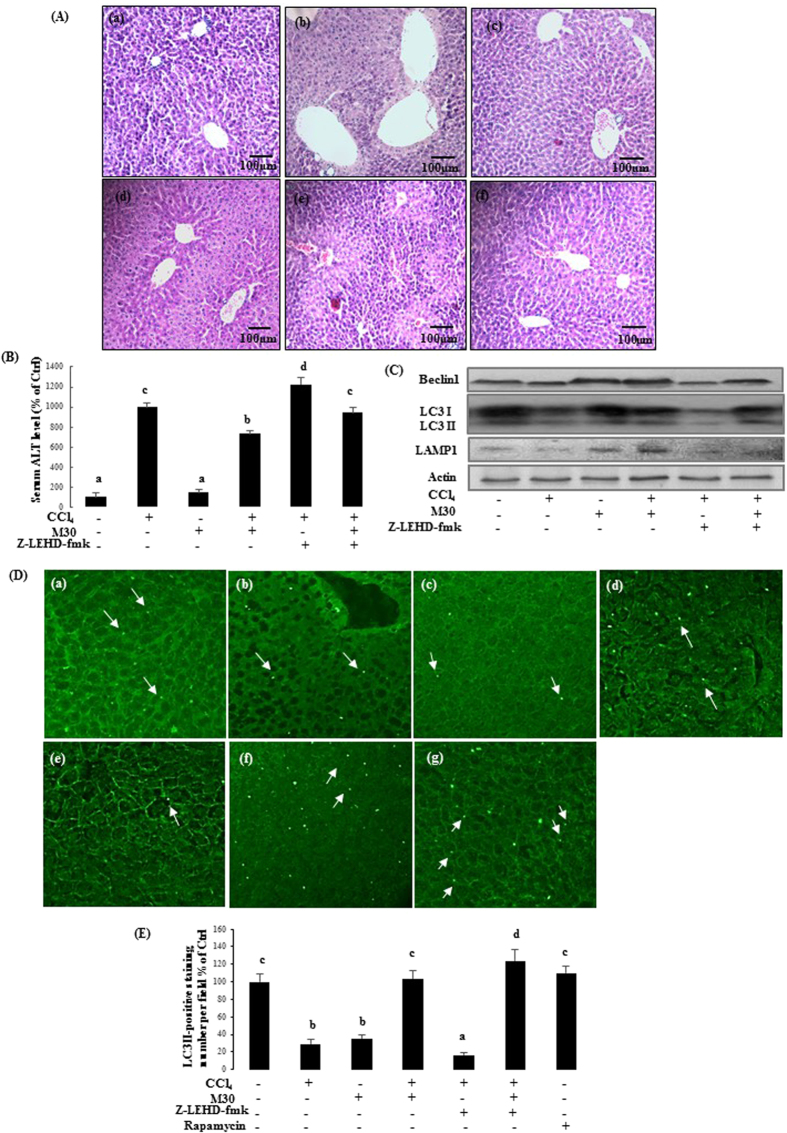
Caspase-9 inhibition markedly suppressed autophagy and exacerbated CCl_4_-triggered acute liver injury in mice. H&E staining of mice liver on (a) Control, (b) CCl4 (75 μl/kg), (c) M30 (5 mg/kg), (d) CCl4 + M30, (e) CCl4 + z-IETD-FMK (3 mg/kg), and (f) CCl4 + M30 + z-IETD-FMK groups (Mag. = 200x) (**A**), as well as the level of serum ALT (**B**). Beclin 1, LC3II and LAMP1 protein expression levels were tested after the injection of z-IETD-FMK (3 mg/kg) (**C**). Immunofluorescence staining of mice liver on (a) Control, (b) CCl4 (75 μl/kg), (c) M30 (5 mg/kg), (d) CCl4 + M30, (e) CCl4 + z-IETD-FMK (3 mg/kg), and (f) CCl4 + M30 + z-IETD-FMK groups (**D**) and statistical analysis shown in (**E**). LC3 aggregation was quantified under fluorescence microscope after immunostaining with LC3B antibody followed by second antibody conjugated with FITC. Bright green dots indicated by arrows are under the field of microscope. Data presented are expressed as Mean ± SD (n = 6) and experimental groups marked by different letters represent significant differences between groups at *p* *<* *0.05* (e.g. a and b mean a statistical significant differences between each other).

For the *in vivo* study, liver sections of CCl_4_-treated mice exhibited hepatic necrosis and inflammation surrounding the centrilobular veins. The necroinflammatory condition further worsened when the mice were co-treated with z-LEHD-fmk (3 mg/kg) suggesting that caspase-9 inhibitor exacerbated CCl_4_-induced hepatic cellular damage. Post-treatment with M30 (5 mg/kg) significantly reduced the areas of necrosis triggered either by CCl_4_ or co-treatment with z-LEHD-fmk. It should be noted that M30 injections alone did not show any histopathological changes in the liver (Fig. 2A). In addition, serum ALT level was remarkably increased after treatment with CCl_4_, and was significantly up-regulated by z-LEHD-fmk, which indicated that inhibition of caspase-9 aggravated acute liver injury. Post-treatment with M30 after CCl_4_ reduced serum ALT level to some extent, suggesting the ameliorative effect of M30 in CCl_4_ plus z-LEHD-fmk induced hepatic injury in mice (Fig. 2B). The protein expression of autophagy markers Beclin1, LC3II and LAMP1 were also tested, which showed similar trends with their corresponding counterparts in HepG2 cells (Fig. 2C), suggesting that autophagy was induced in a caspase-9 dependent manner. The immunostaining result with LC3II antibody was shown in Fig. 2D, and statistical analysis was shown in Fig. 2E. Bright green dots suggested the early stage of LC3 lipidation/aggregation in autophagosomes, as indicated by arrows, which exhibited a similar trend with that of Western blotting results in the liver tissues.

### Inhibition of caspase-9 mitigated HIF-1α-dependent oxidative stress triggered apoptosis

**Figure 3:**
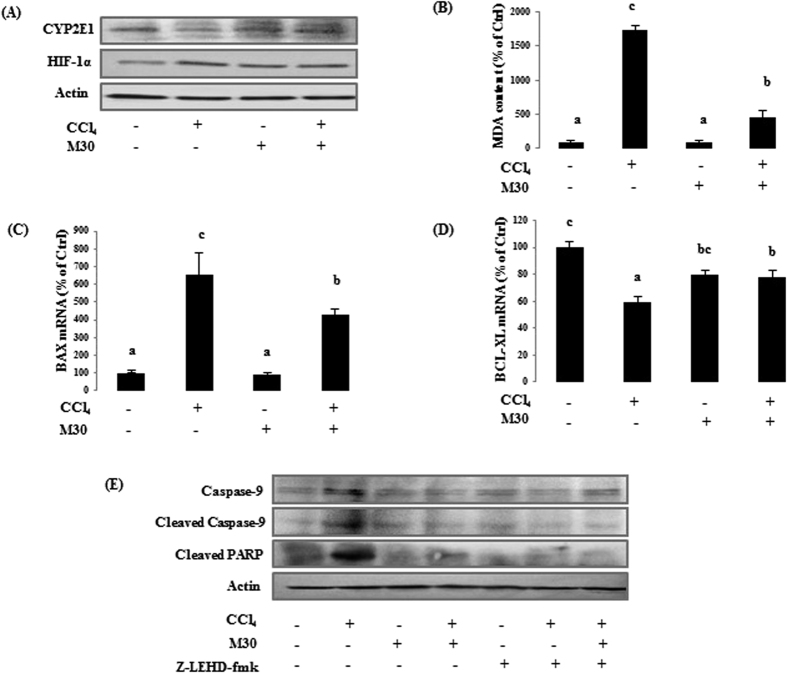
Inhibition of caspase-9 attenuated apoptotic cell death mediated by Hif-1α dependent oxidative stress in HepG2 cells. CYP2E1 and Hif-1α protein level (**A**), formation of MDA (**B**), BAX (**C**) and BCL-XL (**D**) mRNA levels, and caspase-9 and cleaved PARP protein levels (**E**) were evaluated in HepG2 cells. Data presented are expressed as Mean ± SD (n = 6) and experimental groups marked by different letters represent significant differences between groups at *p* *<* *0.05* (e.g. a and b mean a statistical significant difference between each other).

In HepG2 cells, CYP2E1 level was sharply down-regulated by CCl_4_ as a self-defense mechanism to limit the effect of hepatotoxicity, and it was reversed by M30. On the contrary, CCl_4_ significantly up-regulated the protein level of HIF-1α, suggesting its underlying inhibitory effect on prolyl hydroxylase in HIF-1α degradation system. This effect was reduced when CCl_4_ was co-treated with M30 (Fig. 3A). MDA level also showed the same trend suggesting the ameliorative effect of M30 on lipid peroxidation (Fig. 3B). To determine whether CCl_4_ can further trigger apoptosis, the expression levels of apoptosis-related molecules were examined. CCl_4_ markedly up-regulated the mRNA expression of pro-apoptotic mitochondrial protein BAX, which was reduced when co-treated with M30. The expression of the anti-apoptotic protein BCL-XL was opposite with that of BAX (Fig. 3C,D), suggesting that the attenuation effect of M30 on CCl_4_-triggered mitochondrial dependent apoptosis was partly through enhancing the anti-apoptotic protein. In addition, M30 significantly reduced the levels of the cleaved caspase-9 and apoptosis marker cleaved PARP induced by CCl_4_, suggesting the anti-apoptotic property of M30. Moreover, caspase-9 inhibitor z-IETD-FMK (20 μM) inhibited cleaved caspase-9 expression, further leading to the blockage of the down-stream cleaved PARP expression (Fig. 3E).

**Figure 4:**
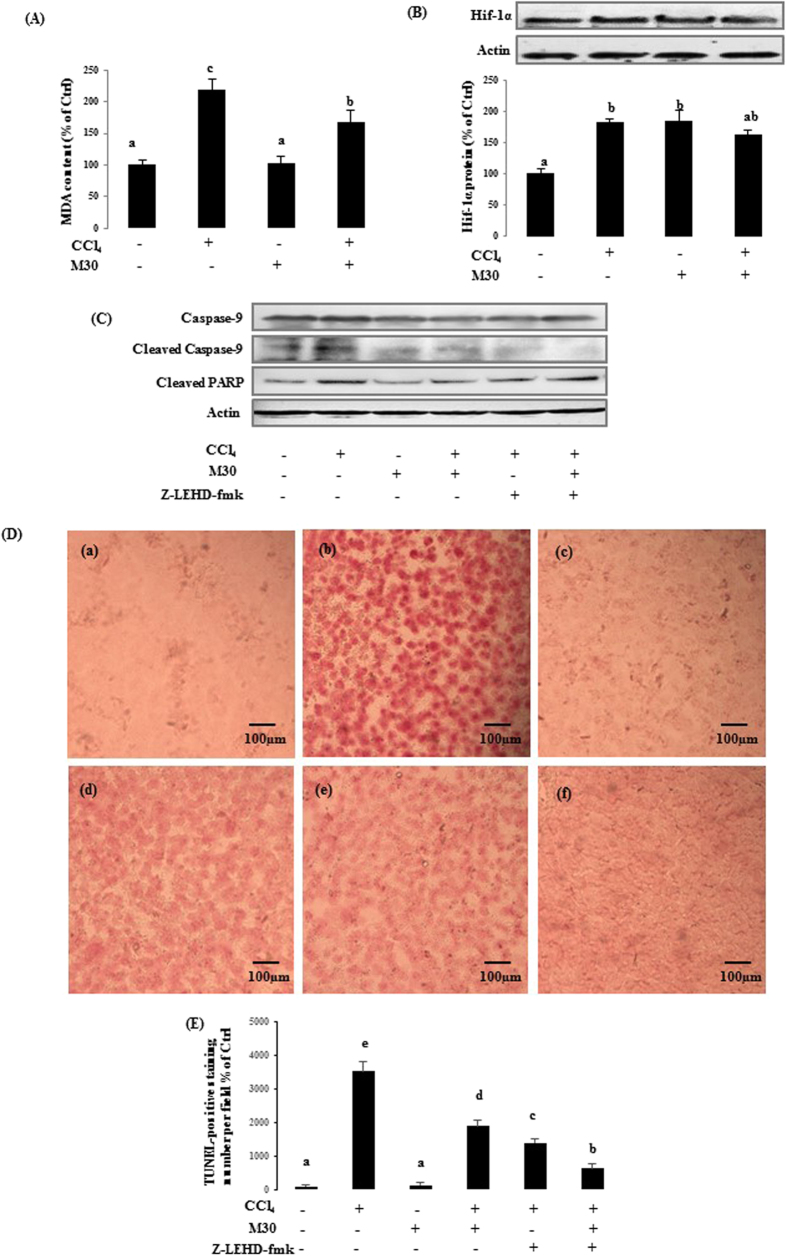
Caspase-9 inhibition reduced Hif-1α dependent apoptosis in mice. The mouse liver MDA level (**A**), Hif-1α protein (**B**), as well as caspase-9 and cleaved PARP protein expressions (**C**) were also measured after CCl_4_, M30 and z-IETD-FMK injection in mice. TUNEL staining was measured in mice liver on (a) Control, (b) CCl_4_ (75 μl/kg), (c) M30 (5 mg/kg), (d) CCl_4_ + M30, (e) CCl_4_ + z-IETD-FMK (3 mg/kg), and (f) CCl_4_ + M30 + z-IETD-FMK groups (Mag. = 200x) (**D**), and statistical analysis as shown in (**E**). Data presented are expressed as Mean ± SD (n = 6) and experimental groups marked by different letters represent significant differences between groups at *p* *<* *0.05* (e.g. a and b mean a statistical significant difference between each other).

To verify the results seen in the *in vitro* model, the related markers in treated C57BL/b6N mice were also examined. MDA (Fig. 4A), HIF-1α protein (Fig. 4B), caspase-9 protein expression, and cleaved PARP protein expression (Fig. 4C) showed similar trends as to their *in vitro* counterpart model. The apoptotic cellular damage was also assessed by TUNEL assay (Fig. 4D), and the statistical analysis is shown in Fig. 4E. TUNEL-positive signals in CCl_4_-injected mice were higher as compared with co-administration of M30 or z-LEHD-fmk groups, indicating the inhibitory effect of M30 and z-LEHD-fmk in apoptosis.

### Inhibition of caspase-9 further elevated necroptosis induced by the pro-inflammatory mediators through TLR4/NF-κB pathway

**Figure 5:**
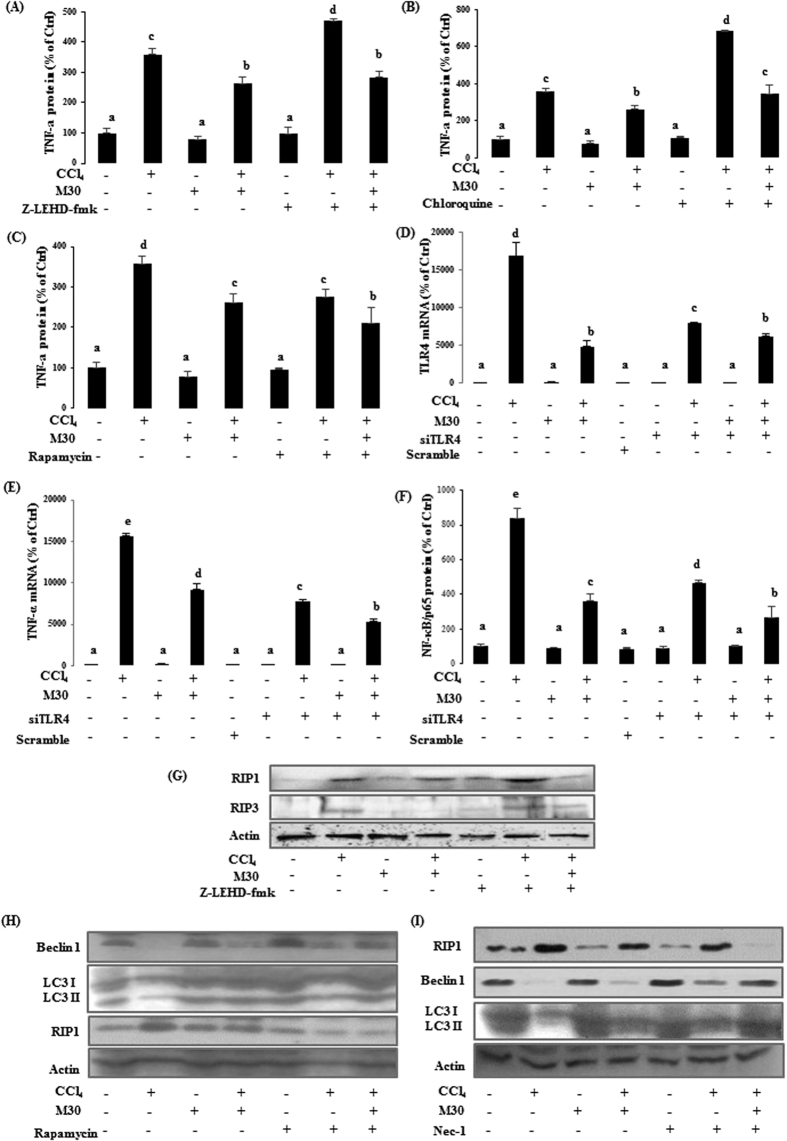
Inhibition of caspase-9 enhanced pro-inflammatory mediator triggered necroptotic cell death in HepG2 cells. Elisa for human TNF-α was tested in HepG2 cells after treatment with z-IETD-FMK (**A**); chloroquine (**B**); and rapamycin **(C)**. TLR4 (**D**), TNF-α (**E**) mRNA, and NF-κB/p65 (**F**) protein levels were also evaluated after the knock down of TLR4 using siRNA. RIP1 and RIP3 protein levels (**G**) were measured using Western blotting. Beclin 1, LC3II and RIP1 protein expression levels were also evaluated after treating rapamycin (20 nM) (**H**) and necrostatin-1 (50 μM) (**I**), respectively. Data presented are expressed as Mean ± SD (n = 6) and experimental groups marked by different letters represent significant differences between groups at *p* *<* *0.05* (e.g. a and b mean a statistical significant difference between each other).

As shown in Fig. 5A–C, the protein level of pro-inflammatory mediator TNF-α was sharply up-regulated after CCl_4_ treatment when compared with the control. After co-treatment with M30, its level was significantly reduced, suggesting that M30 exhibited anti-inflammatory property. Both z-IETD-FMK and autophagy inhibitor chloroquine further accelerated inflammatory process by up-regulating TNF-α levels, while autophagy activator rapamycin exhibited the opposite effect, suggesting that autophagy partly played a beneficial role in suppressing inflammatory process triggered by CCl_4_.

To investigate the role of TLR4 in CCl_4_-triggered inflammation, siRNA knockdown of TLR4 was utilized in HepG2 cells and scramble siRNA was used as a negative control. As shown in Fig. 5D, CCl_4_ remarkably enhanced the TLR4 mRNA expression which was markedly reduced by M30. There was a 60% decrease in the TLR4 mRNA level of CCl_4_ treated cells transfected with 25 nM of siTLR4 when compared to non-transfected CCl_4_ group, suggesting a high transfection efficiency. In addition, increased in mRNA expression of CCl_4_-triggered pro-inflammatory mediator TNF-α and protein level of transcription factor NF-κB/p65 were partially inhibited by TLR4 gene knockdown (Fig. 5E,F). However, the mRNA expression level of antioxidant enzyme CAT and pro-apoptotic protein BAX did not change after TLR4 siRNA transfection in CCl_4_-treated HepG2 cells with or without M30 (data not shown). The above evidence suggested that inhibition of TLR4 only affected the inflammatory effects, but not the oxidative nor the apoptosis effects which were triggered by CCl_4._ In addition, M30 also significantly attenuated CCl_4_-induced inflammatory effects by inhibiting TLR4.

As a pleiotropic cytokine, TNF-α plays a key role in inflammation induced by infection or tissue injury, which can further trigger necroptosis by activating necroptotic markers RIP1 and RIP3^13^. Interestingly, co-treatment with z-IETD-FMK further promoted RIP1 and RIP3 levels (Fig. 5G), suggesting that inhibition of caspase-9 significantly enhanced necroptotic process induced by pro-inflammatory mediators.

To investigate the interrelation of autophagy and necroptosis, autophagy inducer rapamycin and necroptosis inhibitor necrostatin-1 were used. As shown in Fig. 5H, the levels of autophagic markers Beclin1 and LC3II in CCl_4_-treated group were significantly enhanced after the co-treatment with rapamycin. While RIP1 expression was sharply reduced, suggesting that the enhancement of autophagy prevented CCl_4_-triggered necroptosis. On the contrary, necroptosis inhibitor necrostatin-1 markedly decreased RIP1 expression after co-treated with CCl_4_, while autophagic markers Beclin1 and LC3II levels were markedly elevated, which also indicated that inhibition of necroptosis further enhanced autophagy (Fig. 5I).

**Figure 6:**
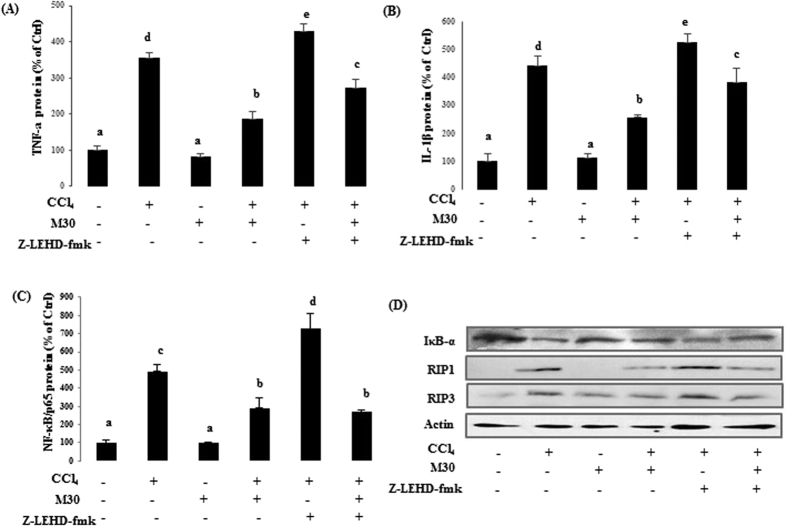
Caspase-9 inhibition remarkably elevated necroptotic markers induced by inflammation in mice. In mouse liver, TNF-α (**A**) and IL-1β (**B**) levels, as well as DNA-binding activity of NF-κB (**C**) were detected by ELISA. Protein levels were also measured by western blotting including total IκB-α protein expression in cytosol, RIP1 and RIP3 in mouse liver (**D**). Data presented are expressed as Mean ± SD (n = 6) and experimental groups marked by different letters represent significant differences between groups at *p* < *0.05* (e.g. a and b mean a statistical significant difference between each other).

In CCl_4_-injected mice and post-treated with M30 showed markedly reduced pro-inflammatory mediators TNF-α and IL-1β levels. Co-treatment of CCl_4_ and caspase-9 inhibitor z-IETD-FMK (3 mg/kg) further increased TNF-α and IL-1β levels, which was down-regulated by post-treatment with M30 (Fig. 6A,B). A similar trend was also observed for the master inflammatory regulator, nuclear transcription factor NF-κB (Fig. 6C), indicating that the inhibition of caspase-9 further aggravated inflammatory process by up-regulating pro-inflammatory mediators and NF-κB. CCl_4_ injury induces NF-κB activation via an IKK-dependent degradation of IκB-α pathway^14^. It was shown that the inhibitory effect of M30 on NF-κB activity was achieved through the reduction in the degradation of the total IκB-α in the cytosol. The *in vivo* expression of necroptotic markers RIP1 and RIP3 exhibited similar trends to their *in vitro* counterparts (Fig. 6D).

### Inhibition of caspase-9 suppressed autophagy, inhibited apoptosis and elevated inflammation-mediated necroptosis in AML12 cell line

**Figure 7:**
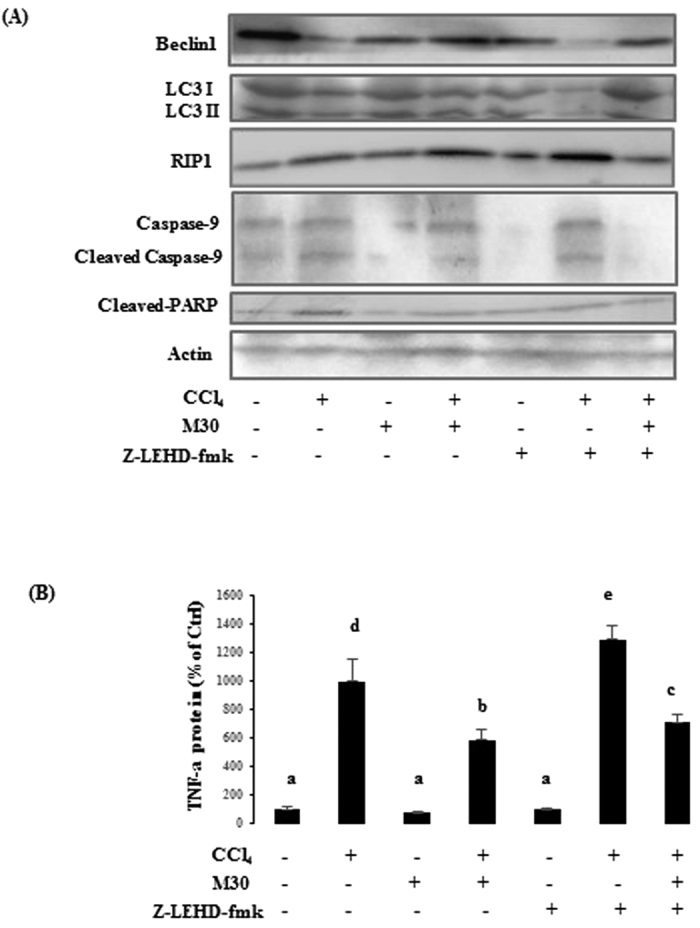
Inhibition of caspase-9 further suppressed autophagy, decreased apoptosis and promoted inflammation-triggered necroptosis in AML12 cells. Protein expressions were also tested by Western blotting including Beclin 1, LC3II, RIP1, caspase-9 and cleaved PARP (**A**). TNF-α protein level was also evaluated using ELISA (**B**). Data presented are expressed as Mean ± SD (n = 6) and experimental groups marked by different letters represent significant differences between groups at *p* *<* *0.05* (e.g. a and b mean a statistical significant difference between each other).

In AML12 cells, the markers for autophagy, apoptosis, inflammation, necroptosis were also tested, including autophagy-modulated proteins Beclin1 and LC3II, apoptotic regulators caspase-9 and cleaved PARP, and necroptosis marker RIP1 (Fig. 7A), which exhibited similar results as the HepG2 cells. In addition, the pro-inflammatory mediator (TNF-α) was also measured by ELISA, showing down-regulation after post treatment with M30 or M30 with caspase-9 inhibitor z-IETD-FMK (Fig. 7B).

## Discussion

In the present study, *in vitro* and *in vivo* experiments were carried out by treating caspase-9 inhibitor z-IETD-FMK to HepG2 cells, AML12 cells and C57BL/b6N mice. The results suggested that inhibition of caspase-9 further suppressed autophagy, implying that caspase-9 may partly act as an autophagy regulator to mediate hepato-protective autophagic process. This is the first study to demonstrate the role of caspase-9 in a cross-link between autophagy and apoptosis, as well as autophagy and inflammation in acute hepatic injury. This finding presents a potential therapeutic target in the liver diseases.

Autophagy induced by the system itself can act as a self-defense mechanism and M30 therapeutic treatment played a protective role in this CCl_4_-induced acute liver injury model. Caspase-9 was involved in the autophagic process of CCl_4_-induced acute liver injury. When caspase-9 inhibitor z-IETD-FMK was added, this further aggravated CCl_4_-induced cell death. The cell viability after adding caspase-9 inhibitor z-IETD-FMK had a similar trend with autophagy inhibitor chloroquine, but it was opposite that of autophagy activator rapamycin. Thus, it is proposed that caspase-9 inhibitor may serve as an autophagy inhibitor to suppress autophagic process. CCl_4_ significantly down-regulated autophagic markers Beclin1, ATG5, LC3II and LAMP1, which were up-regulated by M30, indicating that therapeutic intervention with M30 can achieve the beneficial effects through activation of autophagic process to ameliorate the cellular damage caused by CCl_4_. Interestingly, the expression of autophagic markers Beclin1, ATG5, LC3II and LAMP1 were further down-regulated by z-IETD-FMK, which indicated that the inhibition of caspase-9 further prevented autophagic process to some extent. This suggested that caspase-9 can be considered as an autophagy mediator to trigger hepato-protective autophagy as well as a therapeutic intervention together with M30 to remove the damaged proteins and injured organelles to promotee cell survival. The LC3II staining used in our study to demonstrate autophagy was less optimum since fluorescent staining can give some false positive. TEM demonstrating double lysosomal membrane is considered the optimum technique^15^, and because of this, it imposed some limits in the interpretation our data.

For the involved signaling pathway, a main phosphorylation site Thr125 of caspase-9 was investigated. It has been reported that phosphorylation of caspase-9 at Thr125 restrains apoptosis by blocking the ability of cytochrome c released from mitochondrial to cytoplasm to induce caspase-3^16^. In addition, caspase-9 phosphorylation inclines to be under the control of growth/survival factor signaling as well as being responsive to cellular stresses^17^. In human cells, phosphorylation of caspase-9 at Thr125 is catalyzed by CDK1- cyclin B1 to attenuate apoptotic cell death during mitotic arrest. Interestingly, ERK1⁄ 2 can also target this site to initiate growth factor signaling. Additionally, DYRK1A can also participate in the process of the phosphorylation of caspase-9 at Thr125 during development^18^. This study demonstrated that the autophagy marker Beclin1 expression exhibited a similar trend with phosphor-caspase-9 (Thr125), implying that cytoprotective autophagy can be partly achieved through phosphorylating caspase-9 Thr125 site. Caspase-9 Thr125 was directly phosphorylated by ERK1/2, and likely to play a crucial role in inducing hepato-protective autophagy, and ERK1/2 inhibitor PD98059 reduced caspase-9 phosphorylation at Thr125 as well as autophagy marker Beclin1 expression level. For the specific mechanism, Martin^19^ demonstrated that Thr125 can be directly phosphorylated by ERK, which interacts with caspase-9 through a docking domain located in caspase-9 N-terminal CARD. During this process, Arg10 in caspase-9 is required to interact with Asp160 within a 157TTCD160 motif that is present in ERK, but not in c-Jun N-terminal kinase or p38^19^. As a result, caspase-9 Thr125 site was firstly phosphorylated by ERK1/2, and likely to induce hepato-protective autophagy to inhibit apoptosis and switch on self-defense cell survival process in response to CCl_4_-triggered acute liver damage. The exact mechanism of how phosphorylation of caspase-9 Thr125 triggered autophagy is still not clear and further studies are required to clarify the signaling process.

For apoptosis, CCl_4_ firstly was metabolized by CYP2E1 to generate CCl_3_*, which reacted with oxygen to form CCl_3_OO*, and eventually caused lipid peroxidation catalyzed by CYP2E1, which further contributed to the HIF-1α accumulation Moreover, HIF-1, a complex of HIF-1α and ARNT, has also been demonstrated to activate the transcription factor of p53 to transcribe pro-apoptotic protein BAX, which functions at the mitochondrial membrane to promote release of cytochrome *c*^20^. Then, cytosolic cytochrome *c* interacts with the apoptotic protease-activating factor-1 (APAF-1) and converts procaspase-9 to cleaved caspase-9, finally leading to apoptosis^21^. In the present study, the oxidative stress induced by CCl_4_ caused HIF-1α accumulation, which further activated pro-apoptotic protein BAX and converted procaspase-9 to cleaved caspase-9, ultimately causing apoptotic cell death. M30 attenuated CCl_4_-triggered apoptosis mainly through reducing the upstream oxidative stress, as well as decreasing the expression level of pro-apoptotic protein BAX and increasing anti-apoptotic protein BCL-XL. Apoptosis was also inhibited by caspase-9 inhibitor z-IETD-FMK, which was characterized by the down-regulation of apoptotic marker cleaved PARP.

Even though the apoptotic process was decreased, the total number of cell death unpredictably increased after co-treatment with CCl_4_ and z-IETD-FMK. This may be attributed to the aggravating effect of z-IETD-FMK on inflammation-triggered necroptosis by further up-regulating NF-κB level. It implied that caspase-9 dependent autophagy played a protective role in CCl_4_-triggered inflammation through the inactivation of NF-κB, which served as an important inflammatory regulator to inhibit autophagy and promote delayed programmed cell death. Schlottmann^22^ also demonstrated that the inhibition of NF -κB prevents the increase of inflammatory cells and contributes to the resolution of inflammation^22^. Autophagy can also regulate a number of important immune responses, including clearance of intracellular bacteria, antigen presentation, and the regulation of cytokine production and secretion. Harris^23^ illustrated that autophagy exerts negative feedback on inflammation through digestion of pro-inflammatory mediators by autophagosome^23^. In this study, both z-IETD-FMK and autophagy inhibitor chloroquine further contributed to enhance the inflammatory process by up-regulating pro-inflammatory TNF-α level while the autophagy activator rapamycin exhibited the opposite effect indicating that autophagy acted as a positive regulator to attenuate CCl_4_-triggered inflammatory process in HepG2 and AML12 cells. Similar results of pro-inflammatory mediators TNF-α and IL-1β were also observed after the administration of the caspase-9 inhibitor z-IETD-FMK to mice. Therapeutic treatments such as multifunctional M30 significantly down-regulated the levels of pro-inflammatory mediators which may be due to the activation of the caspase-9 regulated autophagic process to attenuate CCl_4_-triggered inflammation either partly through the reduction of NF-κB activity, or direct digestion of pro-inflammatory mediators.

For the upstream inflammatory pathway, TLR4/MyD88 signaling was investigated. Roh^24^ demonstrated that TLR4/MyD88 plays an essential role in mediating hepatic inflammation and hepato-mitogen expression in liver disease^24^. In CCl_4_-triggered acute liver injury model, our group was the first to report that M30 reduced CCl_4_-induced hepatic inflammation partly through inhibition of TLR4/MyD88/NF-κB signaling pathway by using both *in vivo and in vitro* siTLR4 knockdown models. Our results also showed that only pro-inflammatory mediators were partially inhibited by TLR4 gene knockdown, indicating its fundamental role in initiating the hepatic inflammation. The development of either oxidative stress or apoptosis was not affected. Thus, after administration of CCl_4_, it was firstly catalyzed by CYP2E1 to generate reactive oxygen species CCl_3_* and CCl_3_OO*, which triggered TLR4/MyD88, and further activated key transcription factor NF-κB by degrading cytosolic IκB-α, leading to the transcription of the pro-inflammatory mediators such as TNF-α and IL-1β. Tao^25^ reported that Dioscin exerted its anti-inflammatory properties via the reduction of the expression levels TLR4 level and downstream proteins including MyD88, MAPKs, NF-κB, and AP-1 in dose dependent test, which meant that both MAPKs and TLR4/NF-κB pathways contributed to the development of the inflammation in this system^25^. Nevertheless, in our system, MAPKs-mediated pathway mainly participated in caspase-9 phosphorylation to induce hepato-protective autophagy. Even though MAPKs was involved in inflammatory regulation, TLR4/NF-κB still played the major role in triggering the release of the pro-inflammatory mediators, which can compensate the effects of MAPKs to a great extent.

Necroptosis, also named programmed necrosis, is a form of cell death triggered by the activation of the kinases RIP3, which is generally activated by RIP1^26^. If the conditions are not sufficient to induce apoptotic cell death, pro-inflammatory mediator TNF αinitially activates TNFR1 and in turn recruits RIP1 to form complex I. Subsequently, these proteins combine with other components, and can be located in the cytosol in with complex IIb, which includes RIP1, RIP3, caspase-8 and FADD, further leading to necroptosis^27^. For the As shown in this study, co-treatment of CCl_4_ with caspase-9 inhibitor z-IETD-FMK further enhanced necroptosis RIP1 and RIP3 levels, suggesting that inhibition of caspase-9 accelerated necroptotic process induced by pro-inflammatory mediators. Autophagy and necroptosis have been known to be interconnected. Ye^28^ demonstrated that autophagy negatively regulated necroptosis when caspase-6 was activated in TNFα-treated L929 cells^28^. Additionally, autophagy has been reported to be required for a normal proliferative T-cell response, and deletion of FADD and caspase-8 further enhances autophagy, suggesting that necroptosis inhibition further amplified the autophagic process^29^. Thus, our results suggested the possible beneficial role of caspase-9 dependent autophagy in attenuating inflammation in triggered-necroptotic condition.

In summary, this study presented the possible beneficial role of caspase-9 in autophagic process in CCl_4_-triggered acute liver injury. Apart from the traditional role in apoptosis, caspase-9 can also induce hepato-protective autophagy via the phosphorylation of its Thr125 site by ERK1/2, which effectively mitigated inflammation triggered-necroptosis, leading to the reduction of pro-inflammatory mediator levels such as TNF-α and IL-1β, and finally contributing to the reduction of necroptosis in order to promote cell survival.

## Additional Information

**How to cite this article**: Guo, R. *et al*. Inhibition of caspase-9 aggravates acute liver injury through suppression of cytoprotective autophagy. *Sci. Rep.* **6**, 32447; doi: 10.1038/srep32447 (2016).

## Supplementary Material

Supplementary Information
