# Supplementary material for: Inhibition of caspase-9 aggravates acute liver injury through suppression of cytoprotective autophagy
Source: Sci Rep. 2016 Sep 1;6:32447. doi: 10.1038/srep32447 (PMC5007529; doi:10.1038/srep32447)
Supplement: Supplementary Information [file srep32447-s1.pdf]

Title: Inhibition of caspase-9 aggravates acute liver injury through suppression of cytoprotective autophagy

Authors: Rui Guo[1], Bin Lin*[4], Jing Fei Pan[1], Emily C, Liong[1], Ai Min Xu[2,3], Moussa Youdim[5], Man Lung Fung[1,2], Kwok Fai So[1,2], and George L, Tipoe*[1, 2]

Supplementary Table 1. Primer sequences for quantitative PCR.

Target gene	Direction	Sequence
BAX	Forward	5′-CCCGAGAGGTCTTTTTCCGAG-3′
	Reverse	5′-CCAGCCCATGATGGTTCTGAT-3′
BCL-XL	Forward	5′-AGGCAGGCGACGAGTTT -3′
	Reverse	5′-AGAGTGAGCCCAGCAGAA-3′
TNF-α	Forward	5′-GAGGCCAAGCCCTGGTATG -3′
	Reverse	5′-CGGGCCGATTGATCTCAGC-3′
TLR4	Forward	5′-AGACCTGTCCCTGAACCCTAT -3′
	Reverse	5′-CGATGGACTTCTAAACCAGCCA-3′
GAPDH	Forward	5′-GAAGGTGAAGGTCGGAGTCA -3′
	Reverse	5′-GACAAGCTTCCCGTTCTCAG -3′
